# Pulsed Laser Ablation Characteristics of Light-Absorbing Mask Layer Based on Coating Thicknesses under Laser Lift-Off Patterning Process

**DOI:** 10.3390/mi15060747

**Published:** 2024-06-01

**Authors:** Daehee Hyun, Hee-Lak Lee, Yoon-Jae Moon, Jun-Young Hwang, Seung-Jae Moon

**Affiliations:** 1 Department of Mechanical Convergence Engineering, Hanyang University, Seoul 04763, Republic of Korea; dea_hee@naver.com (D.H.); joylee1112@hanyang.ac.kr (H.-L.L.); myj3235@kitech.re.kr (Y.-J.M.); 2 Korea Institute of Industrial Technology, Ansan 15588, Republic of Korea; jyhwang@kitech.re.kr

**Keywords:** laser ablation, lift-off, laser direct patterning, transparent oxide film

## Abstract

Thin transparent oxide layers are typically patterned for use in electronic products including semiconductors, displays, and solar cells for applications such as transparent electrodes, insulating films, and encapsulation films. Conventional patterning methods have traditionally been used in photolithography and lift-off processes. Photolithography employs the wet development process, which has disadvantages such as potential undercut effects, swelling, chemical contamination, and high process costs. On the other hand, laser ablation, which has the advantages of high accuracy, high speed, a noncontact nature, and selective processing, can be used to pattern thin films. However, absorption in transparent oxide films is usually low. In this study, experiments were conducted to determine the ablation characteristics of mask layers. The factors affecting ablation, including beam radii, fluences, overlap ratios, and coating thicknesses, were examined; and the parameters characteristic of residue-free ablation, namely the ablation threshold, minimum fluence, and minimum ablation linewidth, were also examined. The experimental results revealed that the beam radius was an important parameter in determining the resolutions of transparent films and substrates.

## 1. Introduction

Transparent oxide thin films are widely used in applications such as semiconductors, displays, organic light-emitting devices, and solar cells. There are various processes for the patterning of the transparent oxide thin films [1,2]. The most widely used manufacturing process in the patterning of transparent oxide thin films is the conventional photolithography process [3,4]. In a conventional photolithography process, the target material is typically deposited on a substrate in vacuum by chemical vapor deposition, physical vapor deposition, or atomic layer deposition. A photoresist layer is then spin-coated onto the deposited layer. The residual solvent in the photoresist layer is removed by drying or baking, and the film is exposed to light through a patterned mask. The exposed areas undergo chemical changes in the polymers contained in the resist. Depending on the tone of the photoresist, the exposed and unexposed areas are selectively washed during the development step. This results in a patterned layer of photoresist on the transparent oxide film. An etching process is subsequently implemented, removing the target material that is not protected by the photoresist layer. Finally, the patterning of the transparent oxide film is completed after the removal of remaining photoresist. The lift-off patterning method is often employed when etching damages the device structures or the removal of target material through etching is difficult. In the lift-off patterning method, the target material is deposited after the photoresist is patterned [5,6,7]. By doing so, the target materials in the undesired portion will be deposited on the photoresist layer. After that, the photoresist layer is removed along with the target material on the layer. As a result, the target material only remains in the patterned portion [8,9]. Patterning processes with conventional photolithography and lift-off methods are not easy to implement and have the disadvantages of being complicated and having undercut effects, swelling, high process costs, and chemical contamination [10,11,12]. Recently, laser-based patterning methods such as laser digital patterning [13,14] and direct laser writing [15,16,17,18] have been extensively studied. In laser digital patterning, patterning is achieved with a procedure of nanoparticle ink dispersing, selective laser irradiation, and washing [19]. The laser-irradiated parts are selectively retained in the washing process due to enhanced adhesion. In the direct laser writing patterning method, the laser beam directly ablates the transparent oxide film [20]. In this study, we propose a novel laser-based patterning method which combines the lift-off and laser ablation processes, and which is simple, cheap, and environmentally friendly as compared to pre-existing patterning methods.

**Figure 1:**
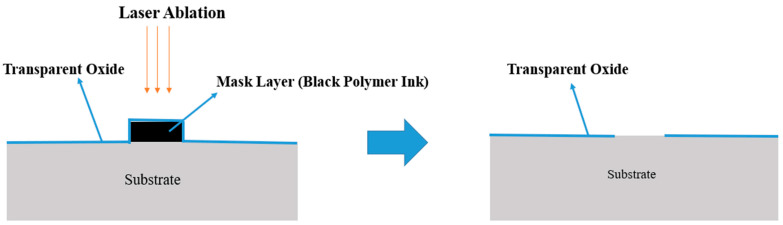
Schematic of the proposed patterning method.

Figure 1 shows a schematic of the proposed laser lift-off patterning method. In the first step of this patterning method, a mask layer is patterned on the substrate through printing. The oxide layer is then coated onto the substrate and the patterned mask layer. Finally, laser is irradiated to ablate the mask layer. The laser ablation of the mask layer also removes the transparent oxide film, which results in a patterned transparent oxide film. In this method, the target of laser ablation is the mask layer and not the transparent oxide film. Thus, visible lasers with lower absorptivity on transparent oxide films can be used for the patterning process, as opposed to the laser direct writing method, which generally requires ultraviolet or infrared lasers, which are relatively expensive and difficult to handle. Moreover, thermal damage or mispatterning of the transparent oxide film due to the laser beam can be avoided if the laser and mask layer are selected to have a higher absorptivity in spectral ranges where the transparent oxide film has a lower absorptivity. This proposed method is simple, cheap, and environmentally friendly because it involves a relatively smaller number of processing steps, uses a cheap visible nanosecond pulsed laser, and does not use environmentally hazardous chemical etchants.

In this article, we have characterized the laser ablation of the mask layer in this newly proposed laser lift-off patterning mechanism using a visible 532 nm laser (F2w-120, Yucoptics, Bohemia, NY, USA) with 16 ns pulse duration. The ablation effect was investigated in terms of beam radius, laser beam spot overlap ratio, and coating thickness. We used the *D*^2^-law to determine the relationship between the ablation linewidth and laser fluence. An optical microscope was used to determine the optimal ablation linewidth and fluence range for residue-free ablation. The relationship between the threshold fluence and the optical properties was investigated using an ultraviolet-visible (UV-Vis) spectrophotometer (Lambda 650, PerkinElmer, Waltham, MA, USA).

## 2. Experiment

**Figure 2:**
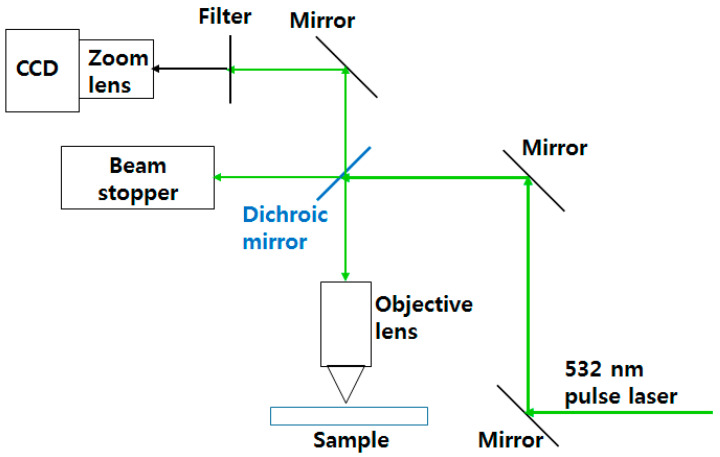
Proposed laser ablation system.

Figure 2 shows an experimental laser system employing a neodymium-doped yttrium aluminum garnet (Nd:YAG) nanosecond pulsed laser with a pulse width of 16 ns and a repetition rate of 30 kHz at a wavelength of 532 nm. A Gaussian laser beam irradiated the sample surface through an infinity-corrected objective lens focused on the sample surface, and the sample was attached to a moving stage. A dichroic mirror was used to transport part of the light reflected from the sample to a charge-coupled device (CCD) camera. A zoom lens was attached to the CCD camera for magnification, and a filter was attached to the zoom lens to block any unwanted exposure. The CCD camera and a moving stage were used to focus the laser beam on the sample surface. The sample was installed through tilting and moving adjustments. The focused 1/*e*^2^ beam radius was approximately 2.2 μm. We used industrial black polymer ink (AlphaChem Co., Ltd., Hwaseong, Republic of Korea) for printing. The ink comprised carbon black, acrylic copolymer, nonionic surfactant, glycerin, triethylene glycol, and deionized water. Its surface tension and viscosity at 23~34 °C were 5.23 dyne/cm and 5.23 cP, respectively. The dimensions of the substrate (Eagle XG, Samsung-Corning, Asan, Republic of Korea) were 24 × 24 × 5 mm^3^ (width × length × thickness). Ultrasonic washing was performed for 10 min in acetone and isopropyl alcohol solutions to remove impurities and organic substances from the substrate. The substrate was then dried at 110 °C for 20 min in an oven to remove any residue. After this, the inks were spin-coated onto the dried substrate at 500, 1000, 2000, and 4000 revolutions per minute to obtain coating thicknesses of 410, 220, 170, and 120 nm, respectively. The average coating thicknesses were measured at three points using an Alpha-Step IQ instrument (Kla-Tencor, Milpitas, CA, USA). Finally, the samples were dried at 110 °C for 15 min on a hot plate to remove any residue.

**Figure 3:**
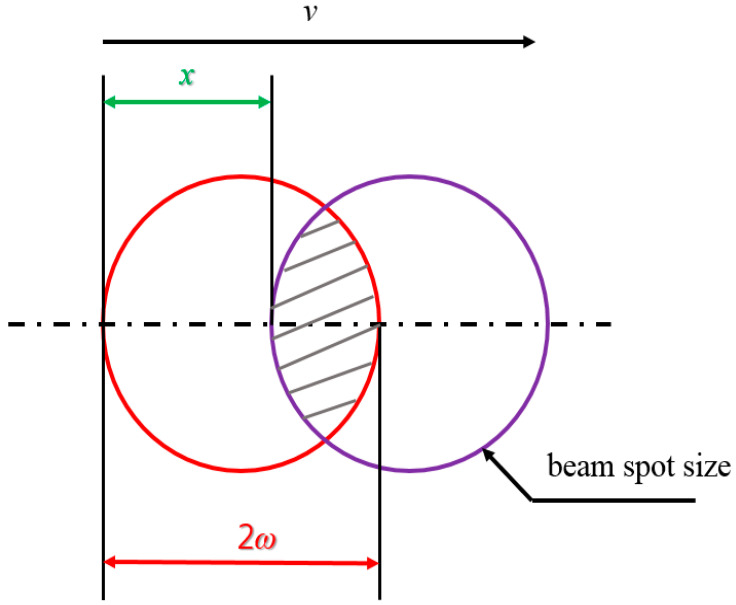
Depiction of the laser overlap ratio.

Figure 3 depicts the overlap between previous and subsequent laser beams. The red circle represents the previous laser beam, the purple circle represents the subsequent laser beam, and the dashed area represents the overlap area. The overlap ratio can be defined based on the laser beam spot size and the moving-stage transport speed as follows [11]:

(1) $$R_{\mathrm{ov}}=\frac{d-x}{d}\times 100(\%),\;x=\frac{v}{f},$$

where *d*, *v*, and *f* denote the laser beam spot size, scanning speed, and laser pulse repetition rate, respectively. Laser beam spot size *d* can be defined as the twice the value of 1/*e*^2^ laser beam radius *w*. The maximum speed of the motorized moving stage speed used in this study was 200 mm/s, limiting the minimum overlap ratio. The minimum overlap ratios were 66 and 83% for laser beam spot sizes of 10 and 20 μm, respectively. The absorbed fluence *F* can be expressed as:

(2) $$F=\alpha \frac{P}{Af},$$

where *α*, *P*, and *A* are the absorbance, laser power, and laser beam area, respectively. The peak fluences were 0.42, 0.84, 1.7, 3.4, and 6.8 J/cm^2^. The absorbed fluence for each case was obtained using Equation (2). The transmittance and reflectance of each sample were measured using the UV-Vis spectrophotometer.

## 3. Results and Discussion

**Figure 4:**
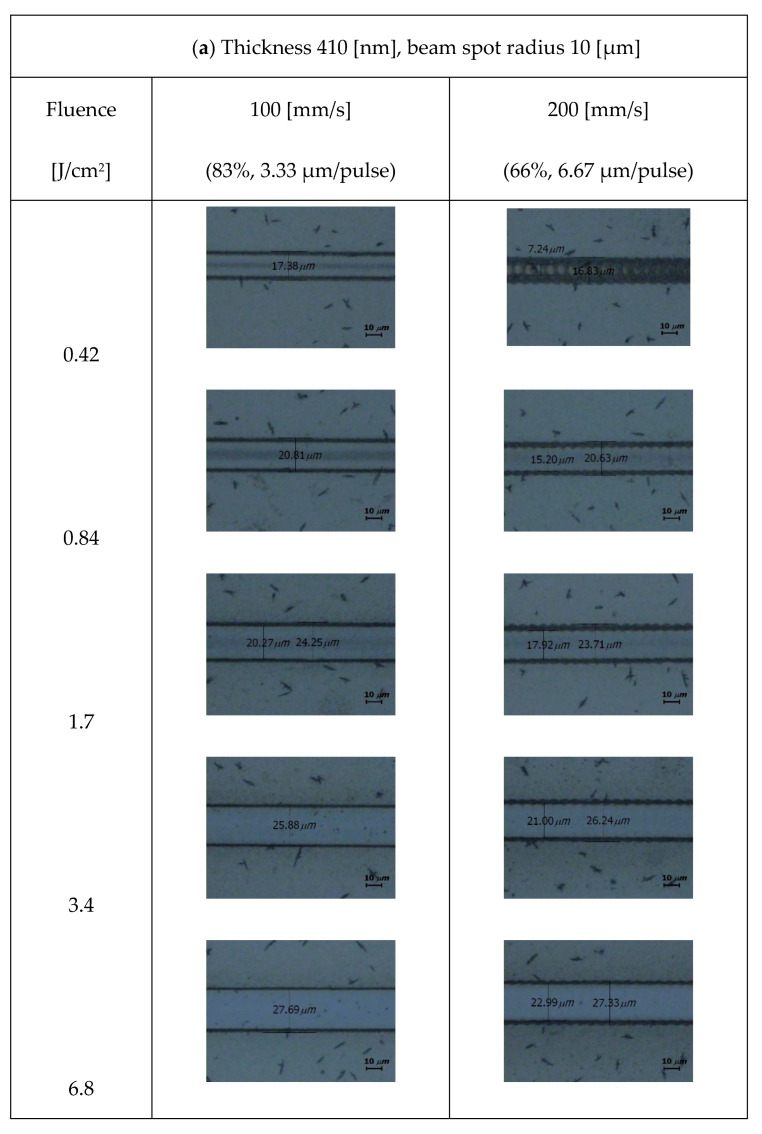

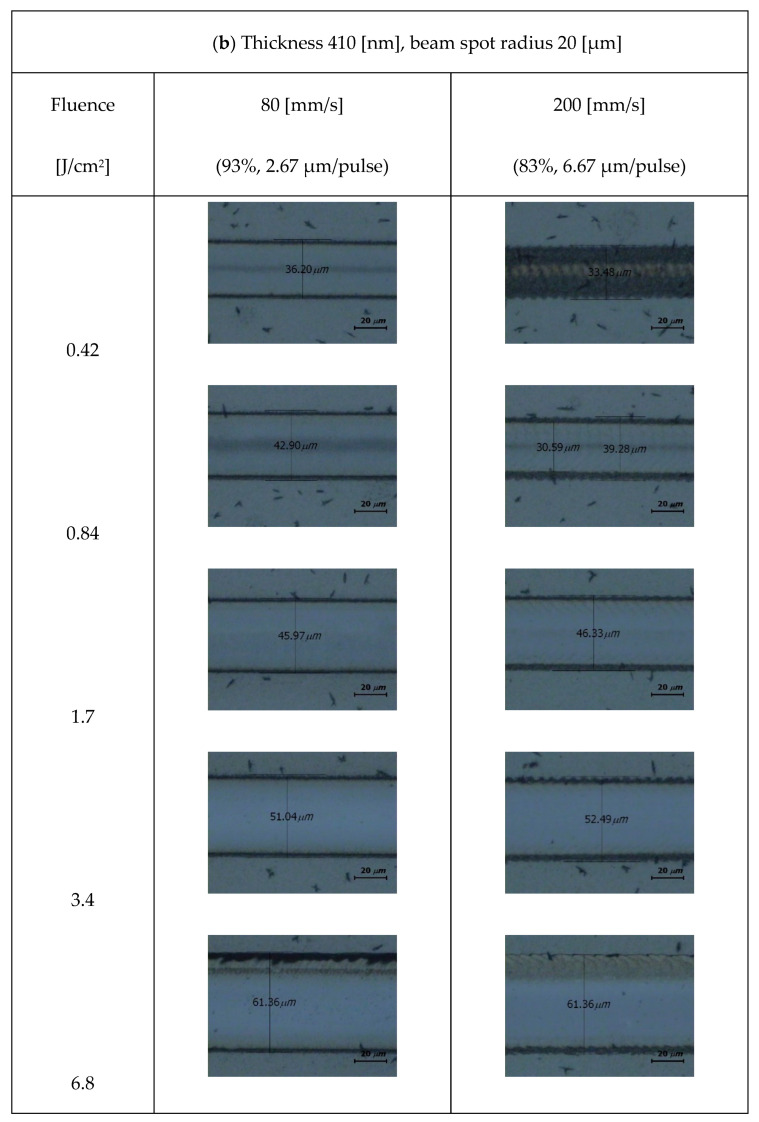
Optical microscopic images of samples with 410 nm coating thickness for various fluence and overlap ratios with beam radii of (**a**) 10 and (**b**) 20 μm. The sporadic black dots are a part of the black polymer ink.

Figure 4 shows the optical microscopic images of a laser-patterned sample with a thickness of 410 nm. Laser beams with radii of 10 and 20 µm were applied under various fluences and overlap ratios. The images showed that the ablation linewidth increased as the fluence increased. Residues were found in the center of the ablated line for fluence below 1.7 J/cm^2^. Regarding beams of identical radii, the residues decreased as the overlap ratios increased.

**Figure 5:**
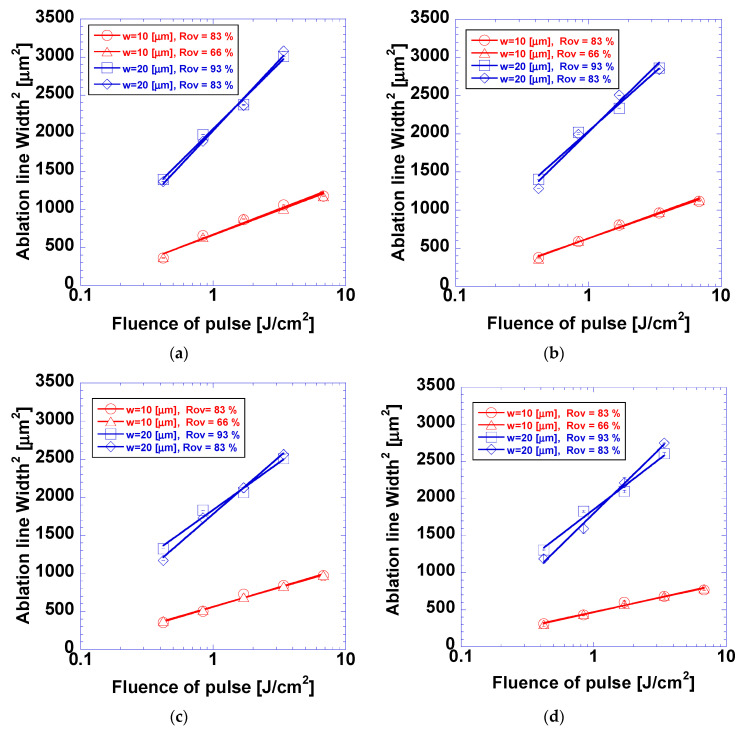
Ablation linewidth of D2-law logarithmic fit with various parameters and coating thicknesses of (**a**) 120, (**b**) 170, (**c**) 220, and (**d**) 410 nm.

Figure 5 shows the squared ablation linewidth for each case. The ablation linewidths were squared so that the *D*^2^-law could be applied. Liu proposed the *D*^2^-law to determine the laser ablation threshold energy of laser beams with Gaussian intensity distribution [21,22]; this law can be expressed as follows:

(3) $$D^{2}=2\omega^{2}\ln\left(\frac{F_{0}}{F_{\mathrm{th}}}\right),$$

where *D*, *ω*, *F*_0_, and *F*_th_ denote the ablation diameter, beam radius, peak fluence, and ablation threshold, respectively. The ablation threshold fluence was determined by extrapolating the linear fitting line of the *D*^2^-law versus ln(*F*_0_) to *D* = 0. Generally, high coefficient of determination (*R*^2^) values exceeding 0.90 indicate the good fitting of the *D*^2^-law. The lowest *R*^2^ value was 0.975 for an overlap ratio of 83% and a beam radius of 20 µm. Note that the case with a laser beam having a radius of 20 μm and fluence of 6.8 J/cm^2^ was not used for *D*^2^-law fitting. The ablation threshold values obtained from the application of *D*^2^-law are listed in Table 1. The overlap ratio did not noticeably affect the ablation threshold fluence. The fitting curves for cases with different overlap ratios but otherwise identical conditions almost coincided with each other. This indicated that the overlap ratio of the laser beam diameter in the scanning direction did not have a significant influence on the ablation linewidth, which was measured in the direction perpendicular to laser scanning. With decreases in the coating thickness, the ablation linewidth increased, and residue-free ablation occurred at lower fluences.

**Figure 6:**
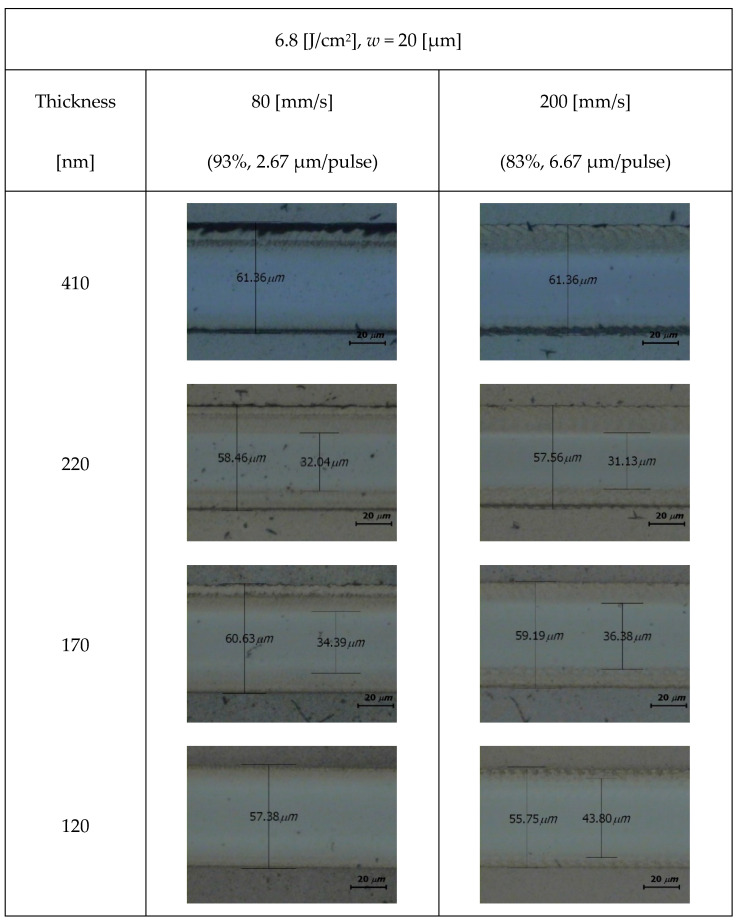
Ablation edges with a laser beam radius of 20 μm, a fluence of 6.8 J/cm^2^, and different coating thicknesses. The sporadic black dots are a part of the black polymer ink.

As shown in Figure 6, there was a significantly incompletely ablated region at the edge, which led to a decrease in the actual linewidth corresponding to the ablation. The increase in the ablation edge was confirmed from Figure 4, where the ablation edge under the laser beam radius of 20 μm and laser fluence of 6.8 J/cm^2^ is much wider than those under the other experimental conditions. The linewidth measured from the outer boundary of the ablation edge was around 60 μm, which was larger than the ablation linewidth in the cases with laser beam radii of 20 μm and lower fluences.

**Figure 7:**
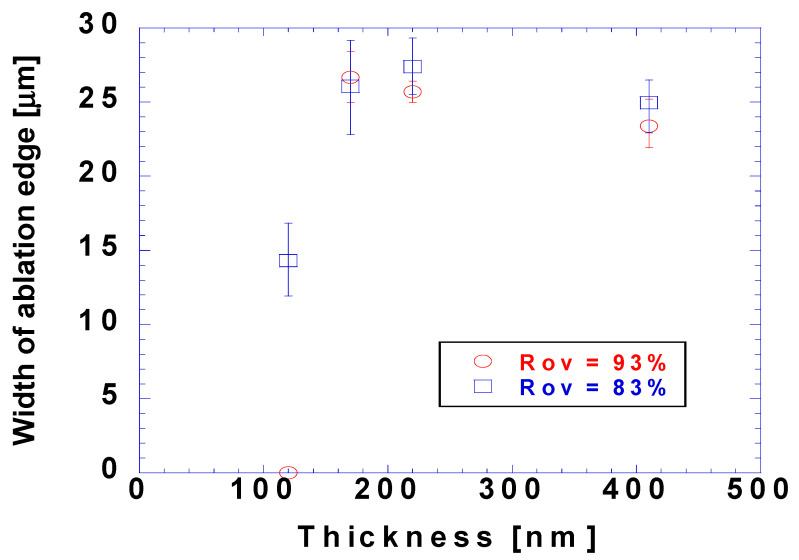
Width of ablation edges according to coating thicknesses with a laser beam radius of 20 μm and a fluence of 6.8 J/cm^2^.

Figure 7 shows the ablation edge width for the cases shown in Figure 6. The ablation edge widths for coating thicknesses of 170, 220, and 410 nm were similar. The influence of the overlap ratio on the ablation edge width was insignificant. The ablation edge width in the case with a coating thickness of 120 nm was noticeably narrower than those in the cases with other coating thicknesses. The influence of the overlap ratio on the ablation edge was significant; the ablation edge shrank to zero in the case with an overlap ratio of 93%. These observations suggested that the rear part of the laser beam with a fluence of 6.8 J/cm^2^ was unsuccessful in the ablation of the black polymer inks; however, additional laser irradiation could alleviate this problem. The failure of the laser beam spot radius of 20 μm under the ablation with a 60 μm linewidth was attributed to the linewidth being three times larger than the 20 μm radius of the 1/*e*^2^ laser beam. Additionally, the rear side of the 60 μm linewidth was 1.5 times wider than that of this 1/*e*^2^ laser beam, which was attributed to the rear side being potentially irradiated by a local laser fluence that was only 1% of the peak fluence. Nevertheless, further studies are required to determine the cause of these unprecedented ablation edges. The absorbance of the target material is an important parameter for laser and target material interactions. A part of the laser energy is reflected at the surface or transmitted through the target material, whereas the remainder is absorbed by the target material. The reflectance, transmittance, and absorbance differ with the material, thickness, and laser wavelength. We measured the transmittance and reflectance using a UV-Vis spectrophotometer and obtained the absorbance as follows:

(4) α=1−τ−ρ

where *τ* and *ρ* are the transmittance and reflectance, respectively.

**Figure 8:**
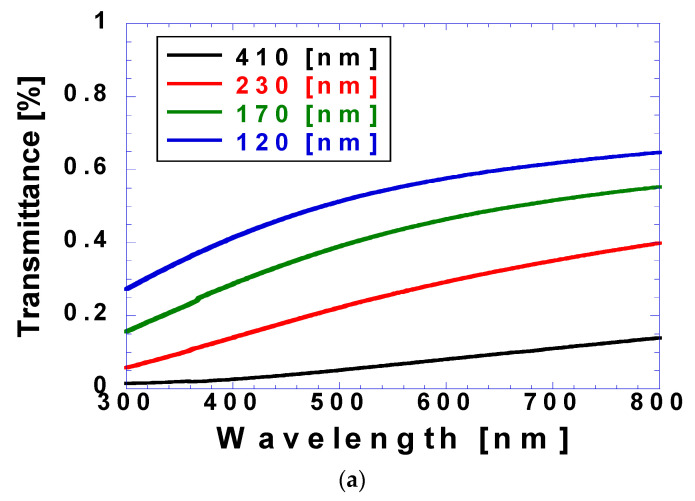

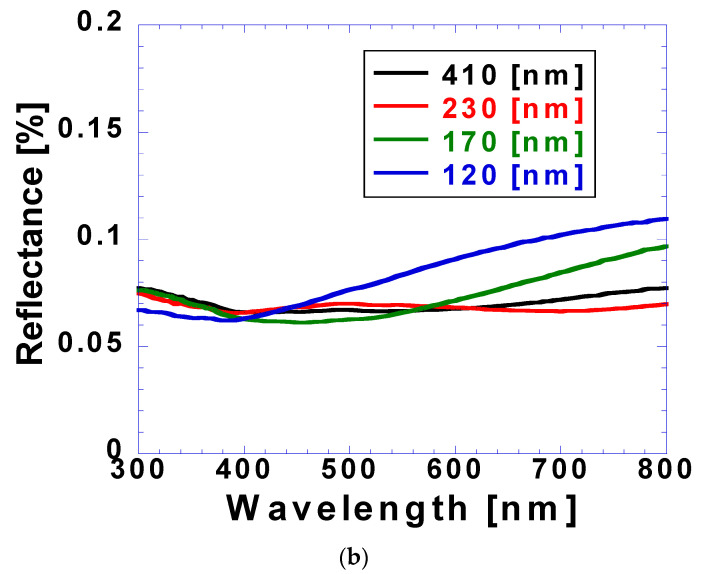
(**a**) Transmittance and (**b**) reflectance obtained using UV-Vis spectrophotometer according to different coating thicknesses.

Figure 8 shows the measured transmittance and reflectance values with respect to coating thickness, with Figure 8a showing that the transmittance decreased with the increasing coating thickness and increased with the increasing wavelength. Specifically, the transmittance of the coating thickness of 120 nm varied from 27% at a wavelength of 300 nm to 65% at a wavelength of 800 nm. In contrast, the transmittance of the coating thickness of 800 nm varied from 1.5% at a wavelength of 300 nm to 14% at a wavelength of 800 nm. Figure 8b shows that unlike transmittance, reflectance (generally within 6–8%) was relatively independent of coating thickness and wavelength. However, the transmittances at coating thicknesses of 120 and 170 nm deviated from this range beyond wavelengths of 400 and 600 nm, respectively. However, the reflectance value was lower than 11%, and the deviations were negligible compared with those of the transmittance. With increasing coating thickness, the reflectance did not vary significantly, and the transmittance decreased, while the absorbance increased.

Table 2 presents the transmittance, reflectance, and absorbance values at 532 nm for coating thicknesses of 120, 170, 220, and 410 nm. The transmittance values were 53.7, 41.7, 24.7, and 6.1%, and the reflectance values were 8.0, 6.4, 6.9, and 6.6%, respectively. The absorbances were 38.3, 51.9, 68.4, and 87.3% for coating thicknesses of 120, 170, 220, and 410 nm, respectively.

**Figure 9:**
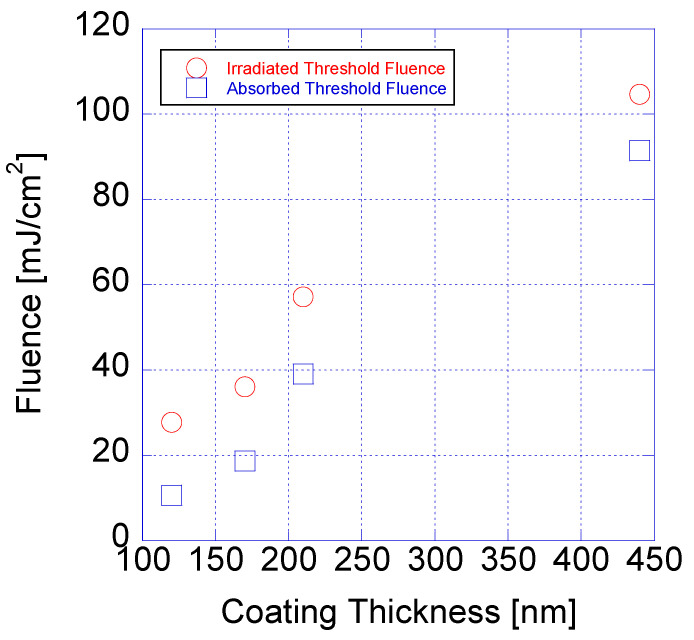
Ablation thresholds based on irradiated and absorbed fluences at different coating thicknesses.

Figure 9 shows the threshold fluence values obtained through *D*^2^-law for the laser spot size of 10 μm and overlap ratio for different coating thickness. For coating thicknesses of 120, 170, 210, and 440 nm, respectively, the threshold fluence values corresponding to the irradiated fluence values were 27.8, 36.1, 57.2, and 104.7 mJ/cm^2^, and those corresponding to the absorbed fluence values were 10.6, 18.7, 39.1, and 91.4 mJ/cm^2^, respectively. The threshold fluence varied significantly depending on the coating thickness, with more energy needed to ablate materials with larger coating thicknesses. As shown in Figure 9, the differences between the threshold fluence values based on the irradiated fluence and absorbed fluence values did not vary significantly; the differences were 17.2, 18.6, 16.1, and 13.3 mJ/cm^2^ for coating thicknesses of 120, 170, 210, and 410 nm, respectively. The difference showed a decreasing trend with the coating thickness, with only minor variations. Although layers with larger coating thicknesses had higher absorbances, the amount of unused energy did not decrease significantly with increasing coating thicknesses because the threshold fluences also increased.

**Figure 10:**
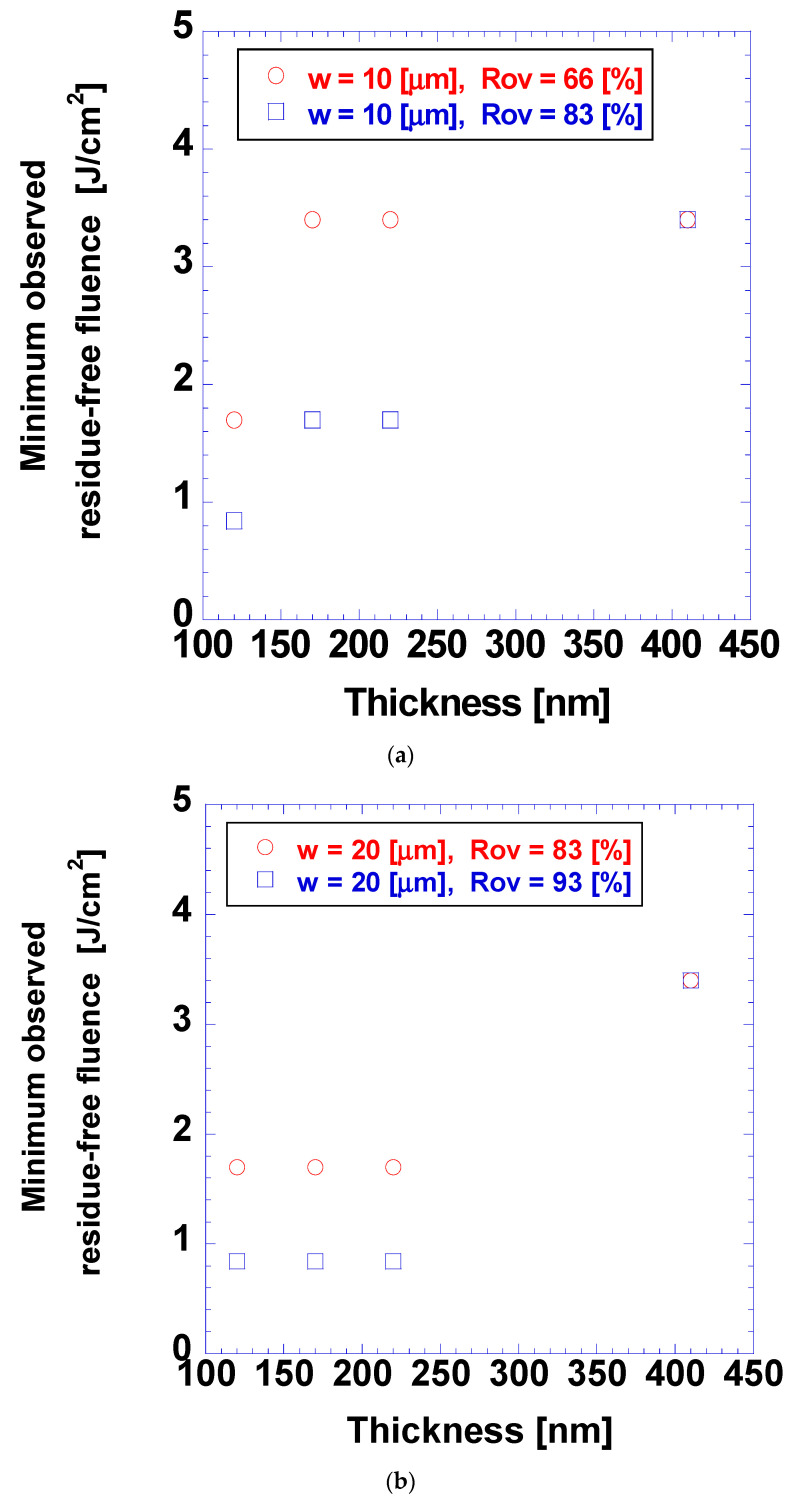
Minimum observed residue-free fluence for different coating thicknesses with overlap ratios of 66 and 83% and laser beam radii of (**a**) 10 and (**b**) 20 μm.

Figure 10 shows the minimum fluence for residue-free ablation. This minimum fluence was determined conservatively as the lowest fluence value with observed residue-free ablation. Generally, a higher fluence was required for residue-free ablation in cases with larger coating thicknesses and lower overlap ratios. Similar trends were observed in cases with beam radii of 10 and 20 µm. The increase in minimum residue-free ablation with the increase in coating thickness was evident from the increased amount of the target material, analogous to the case of the ablation threshold. The influence of the overlap ratios on the minimum residue-free fluence was evident from the translation of the increased overlap ratios to increased irradiated energy per unit area. Noticeably, the cases with the same overlap ratio of 83% and laser beam radii of 10 and 20 μm had similar minimum residue-free fluence values, indicating that the overlap ratio was an important parameter in determining the minimum residue-free fluence.

**Figure 11:**
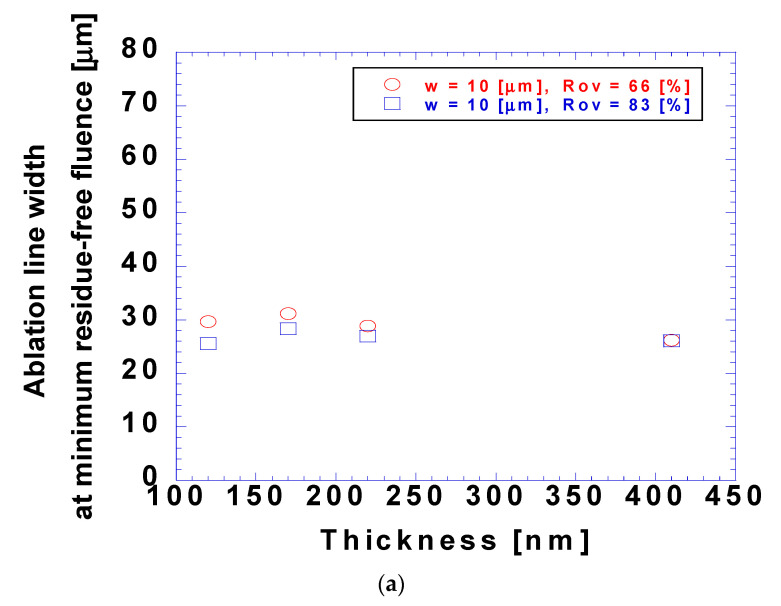

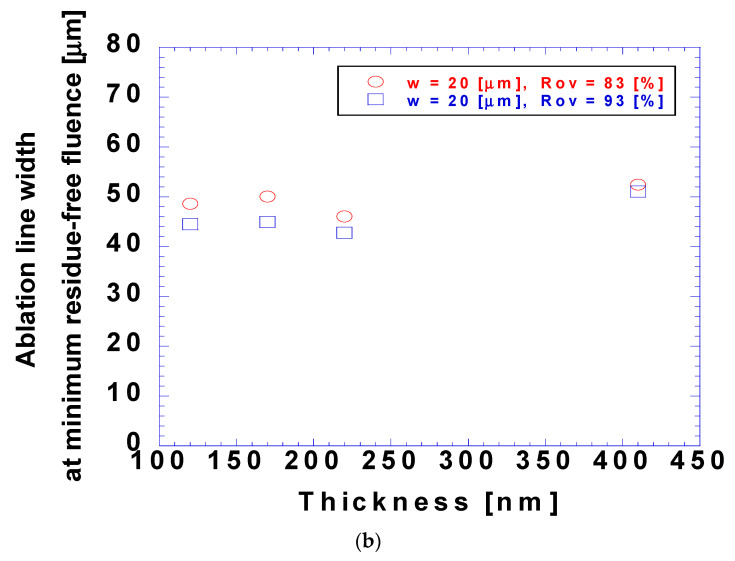
Ablation linewidth at minimum observed residue-free fluence at different coating thicknesses with overlap ratios of 66 and 83% and laser beam radii of (**a**) 10 and (**b**) 20 μm.

Figure 11 shows the ablation linewidth at the minimum fluence for residue-free ablation. Despite the dependence of fluence on coating thickness, the ablation linewidth at the minimum residue-free fluence was relatively independent of this thickness because the threshold fluence increased with increasing coating thickness. Considering that a larger coating thickness did not reduce the amount of unused energy, as shown in Figure 9, the amount of coating could be reduced. In contrast, cases with lower overlap ratios showed a wider ablation linewidth at the minimum residue-free fluence, which could be attributed to the higher fluence required for residue-free ablation. This indicated that although the ablation linewidth and threshold fluence were relatively independent of the overlap ratio, a higher overlap ratio resulted in a better resolution. However, the influence of the overlap ratio on the ablation linewidth at the minimum residue-free fluence was marginal. If the absorbance of the transparent oxide film at visible wavelengths is considered negligible, the overlap ratio would not result in substantial differences in the resolution of laser lift-off patterning. Comparing Figure 11a,b, the laser beam radius was the most influential parameter on the ablation linewidth at the minimum residue-free fluence. The ablation linewidth at the minimum residue-free fluence ranged from 25 to 30 and from 40 to 50 μm when the laser beam radii were 10 and 20 μm, respectively.

**Figure 12:**
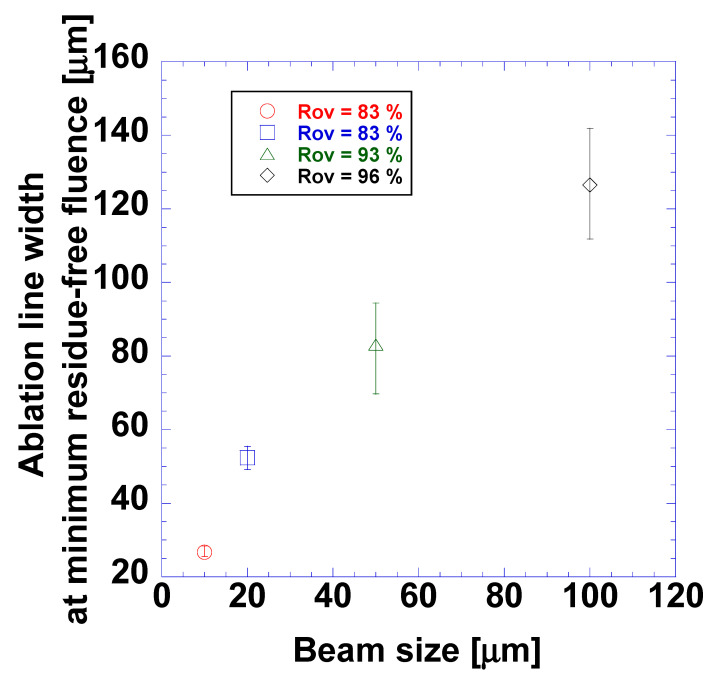
Ablation linewidth at minimum observed residue-free fluence at different beam sizes with various laser beam radii and overlap ratios.

Figure 12 shows the ablation linewidths at the minimum residue-free fluence for beam radii of 10, 20, 50 and 100 µm and high overlap ratios of 83, 83, 93, and 96%, respectively. The laser beam radii were controlled by adjusting the distance between the sample and the objective lens. Clearly, the laser beam radius deeply affected the ablation linewidth at the minimum residue-free fluence. The average values of the ablation linewidth at the minimum residue-free fluence were 27.7, 52.4, 83.1, and 126 μm for laser beam radii of 10, 20, 50, and 100 μm, respectively. The error in the ablation linewidth at the minimum residue-free fluence increased with the laser beam radius. However, because ablation lines slightly wider than the actual printed line did not damage the transparent oxide film and substrate, errors in the ablation of the relatively wider printed lines were less critical. This is an advantage of this method, which utilizes wider ablation lines, over direct laser-patterning methods that use picosecond and femtosecond pulsed lasers [12,17]. The highly increased pulse duration of a nanosecond-pulsed laser results in a much lower intensity and reduces the risk of the transparent oxide film and substrate being damaged. The simplicity of a laser lift-off process that uses nanosecond-pulsed lasers supports its wider accessibility, especially considering that this patterning technique does not use chemical etchants or photomasks.

## 4. Conclusions

We successfully demonstrated laser lift-off patterning using a commercial black polymer ink and a 532 nm nanosecond pulsed laser. Compared to other patterning methods such as photolithography, conventional lift-off patterning, and direct lift-off patterning, this patterning process is much simpler and involves the use of easily accessible equipment. The ablation aspects were investigated for different coating thicknesses in terms of the overlap ratio and beam radius. The *D*^2^-law was used to obtain the ablation threshold from the ablation linewidths. These linewidths were predicted well by this law, according to the ablation threshold, fluence, and beam radius. The minimum fluence for residue-free ablation and the corresponding ablation linewidths were also investigated. The coating thickness affected the threshold fluence and minimum fluence for residue-free ablation. Since both quantities were dependent on the coating thickness, the ablation linewidth at the minimum fluence for residue-free ablation was relatively independent of the coating thickness. The amount of unused energy remained similar regardless of the size of the coating thickness. Additionally, the coating thickness did not affect the laser lift-off process. The overlap ratio did not influence the ablation linewidth or threshold fluence, instead influencing the minimum fluence for residue-free ablation and the corresponding ablation linewidth; the influence of the overlap ratio was marginal compared to that of the laser beam radius. Since the transparent oxide film was not damaged by the operating laser conditions, the laser operating process could be controlled by simply adjusting the radius of the laser beam. In summary, we have introduced a simple patterning method for transparent oxide layers that offers wider accessibility. The introduced laser lift-off patterning method can be applied to other materials using lasers with wavelengths that are transparent to the materials and opaque to commercial polymer inks of other colors.

## Figures and Tables

**Table 1:** The ablation threshold values of the featured cases.

Beam Spot Radius [μm]	Thickness [nm]	Overlap Ratio [%]	Ablation Threshold Fluence [mJ/cm^2^]
10	410	83%	104.7
		66%	104.9
	220	83%	57.2
		66%	57.4
	170	83%	36.1
		66%	35.1
	120	83%	27.8
		66%	29.2
20	410	93%	103.2
		83%	106.2
	220	93%	106.9
		83%	111.7
	170	93%	80.7
		83%	80.6
	120	93%	77.4
		83%	78.7

**Table 2:** Transmittance, reflectance, and calculated absorbance at 532 nm.

Wavelength at 532 nm	Black Polymer Ink		
Thickness [nm]	Transmittance [%]	Reflectance [%]	Absorbance [%]
410	6.1	6.6	87.3
220	24.7	6.9	68.4
170	41.7	6.4	51.9
120	53.7	8.0	38.3

## Data Availability

The data underlying the results presented in this paper are not publicly available at this time but may be obtained from the authors upon reasonable request.

## References

[1] Byeon K.J., Hwang S.Y., Lee H. (2008). Fabrication of nano-hole array patterns on transparent conducting oxide layer using thermally curable nanoimprint lithography. Microelectron. Eng..

[2] Lan J.H., Kanicki J., Catalano A., Keane J., Den Boer W., Gu T. (1996). Patterning of transparent conducting oxide thin films by wet etching for a-Si:H TFT-LCDs. J. Electron. Mater..

[3] Xu G., Liu Z., Ma J., Liu B., Ho S.-T., Wang L., Zhu P., Marks T.J., Luo J., Jen A.K.Y. (2005). Organic electro-optic modulator using transparent conducting oxides as electrodes. Opt. Express..

[4] Budianu E., Purica M., Iacomi F., Baban C., Prepelita P., Manea E. (2008). Silicon metal-semiconductor-metal photodetector with zinc oxide transparent conducting electrodes. Thin Solid Film..

[5] Aleksandrova M., Kolev G., Cholakova I., Dobrikov G., Bodurov G. (2013). Photolithography versus lift off process for patterning of sputtered indium tin oxide for flexible displays. Int. J. Thin Film. Sci. Technol..

[6] Chae J., Appasamy S., Jain K. (2007). Patterning of indium tin oxide by projection photoablation and lift-off process for fabrication of flat-panel displays. Appl. Phys. Lett..

[7] Andrews A.M., Liao W.S., Weiss P.S. (2016). Double-Sided Opportunities Using Chemical Lift-Off Lithography. Acc. Chem. Res..

[8] Mackus A.J.M., Bol A.A., Kessels W.M.M. (2014). The use of atomic layer deposition in advanced nanopatterning. Nanoscale.

[9] Biercuk M.J., Monsma D.J., Marcus C.M., Backer J.S., Gordon R.G. (2003). Low-temperature atomic-layer-deposition lift-off method for microelectronic and nanoelectronic applications. Appl. Phys. Lett..

[10] Venkat S., Dunsky C. (2006). Laser patterning of ITO in flat panel display manufacturing. Photon Processing in Microelectronics and Photonics V.

[11] Shin H., Sim B., Lee M. (2010). Laser-driven high-resolution patterning of indium tin oxide thin film for electronic device. Opt. Lasers Eng..

[12] Risch A., Hellmann R. (2011). Picosecond laser patterning of ITO thin films. Phys. Procedia..

[13] Nam V.B., Shin J., Yoon Y., Giang T.T., Kwon J., Suh Y.D., Yeo J., Hong S., Ko S.H., Lee D. (2019). Highly Stable Ni-Based Flexible Transparent Conducting Panels Fabricated by Laser Digital Patterning. Adv. Funct. Mater..

[14] Nam V.B., Lee D. (2024). Highly transparent and low-voltage-driven soft actuators fabricated by laser digital patterning. Opt. Laser Technol..

[15] Brygo F., Dutouquet C., Le Guern F., Oltra R., Semerok A., Weulersse J.M. (2006). Laser fluence, repetition rate and pulse duration effects on paint ablation. Appl. Surf. Sci..

[16] Chen M.F., Chen Y.P., Hsiao W.T., Gu Z.P. (2007). Laser direct write patterning technique of indium tin oxide film. Thin Solid Film..

[17] Choi H.W., Farson D.F., Bovatsek J., Arai A., Ashkenasi D. (2007). Direct-write patterning of indium-tin-oxide film by high pulse repetition frequency femtosecond laser ablation. Appl. Opt..

[18] Cheng C.W., Lin C.Y. (2014). High precision patterning of ITO using femtosecond laser annealing process. Appl. Surf. Sci..

[19] Nam V.B., Giang T.T., Koo S., Rho J., Lee D. (2020). Laser digital patterning of conductive electrodes using metal oxide nanomaterials. Nano Converg..

[20] Saetang V., Qi H., Smerchit T., Rujisamphan N. (2022). Laser scribing of fluorine-doped tin oxide coated on glass substrate in air and water. Opt. Laser Technol..

[21] Liu J.M. (1982). Simple technique for measurements of pulsed Gaussian-beam spot sizes. Opt. Lett..

[22] Baudach S., Bonse J., Krüger J., Kautek W. (2000). Ultrashort pulse laser ablation of polycarbonate and polymethylmethacrylate. Appl. Surf. Sci..

